# Domestic Correspondence

**Published:** 1889-01

**Authors:** 


### Domestic Correspondence.

To The Editor:

The dentists of New York probably enjoy a more perfect organization than those of any other State. There are eight district societies, holding regular annual and semi-annual meetings, and delegates from these, with its own permanent members, make up the State Society, which meets annually. All dues to the latter are paid by the District Societies, and thus is secured an organization which does not depend upon the chances of voluntary attendance. The government of dentists in their professional relations is entrusted to the State Society, which acts through its delegated representatives. All these societies are organized under due process of law, and at each annual meeting there are certain legal requirements to meet, which obliges them to hold the annual meeting at definite times and places within the bounds of each district.

But the semi-annual meetings are held solely for scientific purposes, and it has been the case that frequently two or more District Societies hold their meetings together. Such a one was the late Syracuse reunion, perhaps the most noteworthy and successful of any since their organization more than twenty years ago. It was eminently fit and proper that such a meeting should be held at Syracuse, for Onondaga County, within whose limits the city is situated, has been the mother of many whose names are held in high honor by dentists. Chapin A. Harris, the father of modern dentistry, was born in this county, and it was also the home of the lamented Amos Westcott. Wm. H. Dwinelle here commenced his honorable professional career, and his warm heart has ever cherished in its warmest corner a love for the home of his early days. Frank Abbott and Albert H. Brockway long ago left Onondaga for a richer field of usefulness in New York, while John S. Marshall strayed to Chicago, and there are others who claim Onondaga as the initial point in their useful lives. All of these who are yet living, if we except Dr. Abbott, were present at the meeting, and their number was swelled by such men as E. T. Darby of Philadelphia, T. W. Brophy of Chicago, J. B. Willmott of Toronto, W. Geo. Beers of Montreal, and W. C. Barrett, of Buffalo. I shall not attempt a report of the meeting; but it may be comprehended that the occasion was one of great interest and profit. Some of the papers read possessed great merit, and it is a source of gratification to know that the best of these were written by practicing dentists. Several addresses were made by invited medical men, but the same interest did not centre in them.

There was a very unusual display of dental goods, the S. S. White Company exhibiting a complete electrical plant. The clinics were especially instructive, a large number of surgical cases being presented. A grand banquet was given by the Fifth District Society, whose guests the other societies were, at which about three hundred were present, and speeches, sentiments and music entertained all until a late hour, while on the same evening the ladies who accompanied the members and visitors were tendered a reception at the hospitable home of Dr. Charles Barnes. Great credit is due the Syracuse dentists who labored so unceasingly to make of the meeting the great success which it proved.

The next semi-annual meeting of the four Societies will be with the Sixth District, at Elmira, a year hence, and as the members of that Society have already commenced the work of preparation, a memorable time is certain. *

---

To the Editor:

The annual meeting of the American Academy of Dental Science was held in Boston on Wednesday, Nov. 14. A number of members of the New York Odontological Society were present, in response to special invitations sent to that body. Gentlemen were also present from Providence, Newport, Salem and other places. In the morning the members of the Academy and their guests visited Cambridge in carriages provided by the former. The day was beautiful, and the ride a delightful one. On reaching Harvard College grounds, the entire party visited several of the College buildings; but the chief point of interest was the Peabody Museum, where Prof. Putnam, who has charge of the department, received the visitors with a warm welcome, and conducted them from room to room, showing and explaining the various specimens as well as possible in the limited time given him. The numerous specimens of crania particularly interested the visitors, who were delighted with what they saw, and also with the genial professor for his kindness and for the cordial reception he gave them.

On their return to Boston, the guests were conveyed to the Algonquin Club-house, a new and magnificent structure with exquisite appointments, which called forth many expressions of admiration. In one of the elegant dining-rooms a bounteous repast was prepared for the party, who, when seated around the long table, did ample justice to the festive occasion.

At 4 P.M., the members of the Academy and their friends assembled at Young's Hotel, where a parlor had been engaged for the annual meeting. Dr. C. P. Wilson, President of the Academy, called the meeting to order, and an hour or more was spent in listening to the reading of annual reports of officers, committees, etc.; after which was an election of officers for the ensuing year. All the officers of the term just expired were re-elected; which seemed good evidence that they were just the right men for their places.

The regular business of the meeting being over, the President introduced Dr. C. E. Francis, of New York, as orator of the occasion.

Dr. F. announced as his subject, "The Achievements and Hopes of our Specialty," in which he referred to early periods of medical practice, the progress of medical science, and the creation of special departments in medicine. He paid a just tribute to the pioneers and teachers of dental art and science, who had done so much to ennoble their calling. He spoke of the grand work done by dentists, and of the many achievements they had accomplished. He complimented the dental colleges, and instructors who had devoted their time and talent in the cause of professional advancement. He looked upon dental societies as the life and strength of our specialty, and referred to the great work they had done.

Dr. Francis dwelt at some length on the relation of dentistry to medicine; considering it a legitimate branch of the mother profession, and fully recognized as such by the American Medical Association and the International Medical Congress. The doctor closed his address by exhorting his hearers to spare no endeavor to honor their chosen specialty; to discharge each and every duty forced upon them with an intelligent will, and thereby sustain the position claimed and conceded as specialists of the great "healing art."

At the close of the address, a vote of thanks was tendered to Dr. Francis, and the meeting adjourned to partake of a banquet in one of the large dining-rooms of the hotel.

The table and appointments were exceedingly fine, and nearly fifty happy gentlemen were comfortably seated. Our Boston friends gave decided evidence of their ability to entertain their guests in a most charming and enjoyable manner. Such a profusion of good things provided for the inner man; such expressions of good and satisfactory feeling all around; such capital after-dinner speeches; and such a flow of complimentary utterances would be difficult to surpass or equal. Rev. Phillips Brooks, the great Boston preacher, who was present, partook of the happy spirit of the occasion, and made one of his characteristic and delightful speeches. After nearly five hours had been spent in this genial way, the party adjourned, with kind adieus and mutual good wishes. *

---

To the Editor:

Will you kindly give place in the I. D. J. to the enclosed correspondence, and oblige, F. A. Levy.

F. J. S. Gorgas, M.D., D.D.S., Orange, December 1, 1888.
Dean Dental Department University of Maryland.

*Dear Sir:* My attention has just been called to the correspondence published by you in the October number of the *American Journal of Dental Science*, much of it dated in November; from which it appears that you have suppressed my answer to yours of November 1, wherein I gave you the full particulars, as requested, of the dropping of the name of the University of Maryland Dental Department from the list of colleges whose diplomas should be recommended to the State Examining Boards, to be received in lieu of an examination.

I further notice, upon page 285 of the same issue of the *American Journal of Dental Science*, in a letter to Dr. T. S. Waters, you say: "I wrote to Dr. Frederick A. Levy, of Orange, N. J., asking him 'if our dental department had been omitted from the list of dental schools by your association, and, if so, for what cause,' etc., etc. He returned no answer to my questions."

By the publication of that letter at this time, and after you had received my answer, before referred to, you could have had no other intention than to place me in an unenviable position, and to mislead the public. It is a very weak cause which descends to these peculiar methods of bolstering itself up.

Kindly let me hear from you, by return mail, whether I can look to you to correct the above errors, and also to publish my answer of November 5th, in the same publications in which appeared the balance of this correspondence?

Very truly yours, Frederick A. Levy, *Secretary.*

[Not hearing from Dr. Gorgas, Dr. Levy wrote the publishers of the *American Journal of Dental Science*, Messrs. Snowden & Cowan; and not hearing from them either, Dr. Levy, in justice to himself, therefore writes us, requesting that we publish the entire correspondence between himself and Dr. Gorgas, which we will gladly do in our next issue.—Ed.]

